# *rpoB* Targeted Loop-Mediated Isothermal Amplification (LAMP) Assay for Consensus Detection of Mycobacteria Associated With Pulmonary Infections

**DOI:** 10.3389/fmed.2018.00332

**Published:** 2018-11-28

**Authors:** Simon Grandjean Lapierre, Michel Drancourt

**Affiliations:** ^1^Aix-Marseille Université, IRD 198, MEPHI, IHU Méditerranée Infection, Marseille, France; ^2^Centre de Recherche du Centre Hospitalier de l'Université de Montréal, Montreal, QC, Canada

**Keywords:** non-tuberculosis mycobacteria, *Mycobacterium tuberculosis*, loop mediated isothermal amplification, nuclear acid amplification assay, pulmonary infection

## Abstract

Loop-mediated isothermal amplification (LAMP) is a nucleic acid method which has been used to identify mycobacteria including *Mycobacterium tuberculosis* in clinical microbiology laboratory and point of care settings. Previously published LAMP protocols for detection of mycobacterial species used conventional specific primer and targeted the 16S rRNA, *gyrB*, and insertion sequence genes. We developed and evaluated a LAMP assay targeting a mycobacterial *rpoB* gene conserved sequence and incorporating degenerate primers. This assay allowed consensus detection of mycobacterial species from pure culture, clinical respiratory tract samples, and mycobacteria growth indicator tube (MGIT) liquid-based culture medium. A panel of twenty mycobacterial species were successfully detected at detection thresholds of 10^2^ CFU/mL and 10^3^ CFU/mL when respectively performed on pure culture suspension or sputum and MGIT broth. The inclusion of degenerate bases in LAMP primers increased the diversity of mycobacterial species identified by the assay without negatively affecting analytical sensitivity. LAMP-based consensus detection of multiple pathogens can be achieved with degenerate primers therefore allowing the design of rapid multi-disease screening assays. Despite high analytical sensitivity, species specificity and the advantageous operational characteristics of LAMP over PCR, challenges such as potential ambiguity in visual interpretation of results and occasional non-specific amplification precludes the implementation of novel LAMP assay in routine diagnostics both in centralized and point-of-care laboratory.

## Introduction

Tuberculosis remains the bacterial disease with the highest mortality worldwide with 1.3 million deaths in 2016 (1). Although less frequent, a growing diversity of non-tuberculous mycobacteria (NTM) are increasingly recognized as lung pathogens. Longevity of immunocompromised populations, chronic pulmonary diseases and increased invasiveness of care and associated nosocomial incidents are factors contributing to this changing epidemiology (2–4). NTM pulmonary infections typically present as non-specific respiratory clinical syndromes in vulnerable populations and require long term and potentially toxic multi-drug therapies which are not routinely prescribed empirically. Rapid diagnosis of mycobacterial disease relies on poorly sensitive and specific staining or targeted nuclear acid amplification tests (NAATs) (5–7). In routine microbiology diagnostic laboratories, species specific mycobacterial NAATs are performed on selected clinical samples with high positive pre-test probability and are therefore tributary of accurate clinical suspicion and optimal communication between clinicians and laboratories. These assays are generally highly sensitive and specific but poorly adapted for rapid screening of pulmonary NTM infections in the face of non-specific respiratory clinical syndromes.

Loop-mediated isothermal amplification (LAMP) is an isothermal NAAT which has been developed and optimized for microbiology diagnostics (8, 9). Through autocycling strand displacement DNA synthesis by a *Bst* DNA polymerase, LAMP technology isothermally synthesizes high amounts of variable size DNA amplification products. LAMP technology has the following advantages: (i) it is highly specific due to a set of four to six primers recognizing six to eight specific DNA regions; (ii) it is independent of thermocycling therefore reducing hands-on and total turnaround time; (iii) it produces large quantities of DNA allowing visual interpretation of results using fluorescent intercalant agents or pH indicators; (iv) it has higher tolerance to inhibitors and is therefore compatible with simpler and faster extraction protocols. These characteristics are well suited for point-of-care diagnosis and LAMP assays have previously been evaluated for diagnosis of mycobacterial and other infectious diseases in this setting (10). Based on these theoretical advantages over standard polymerase chain reaction (PCR)-based assays, several LAMP assays were developed for the rapid detection of mycobacteria in clinical samples. Yet these assays have been limited to the detection of specific mycobacterial species by targeting the 16S rRNA, *gyrB*, and insertion sequence genes (11–13).

We previously introduced the *rpo*B gene as a suitable molecular target for the detection and identification of mycobacteria (14). To increase the spectrum of available tests for the rapid diagnosis of pulmonary mycobacterioses at point-of-care level, we here designed and evaluated a LAMP assay incorporating degenerate *rpoB* gene LAMP primers for consensus detection of mycobacterial species from pure culture, clinical respiratory tract samples and mycobacteria growth indicator tube (MGIT) liquid-based culture medium. This is the first reported LAMP assay to use degenerate primers to achieve consensus detection of multiple pathogens.

## Materials and methods

### LAMP primers design

**Figure 1:**
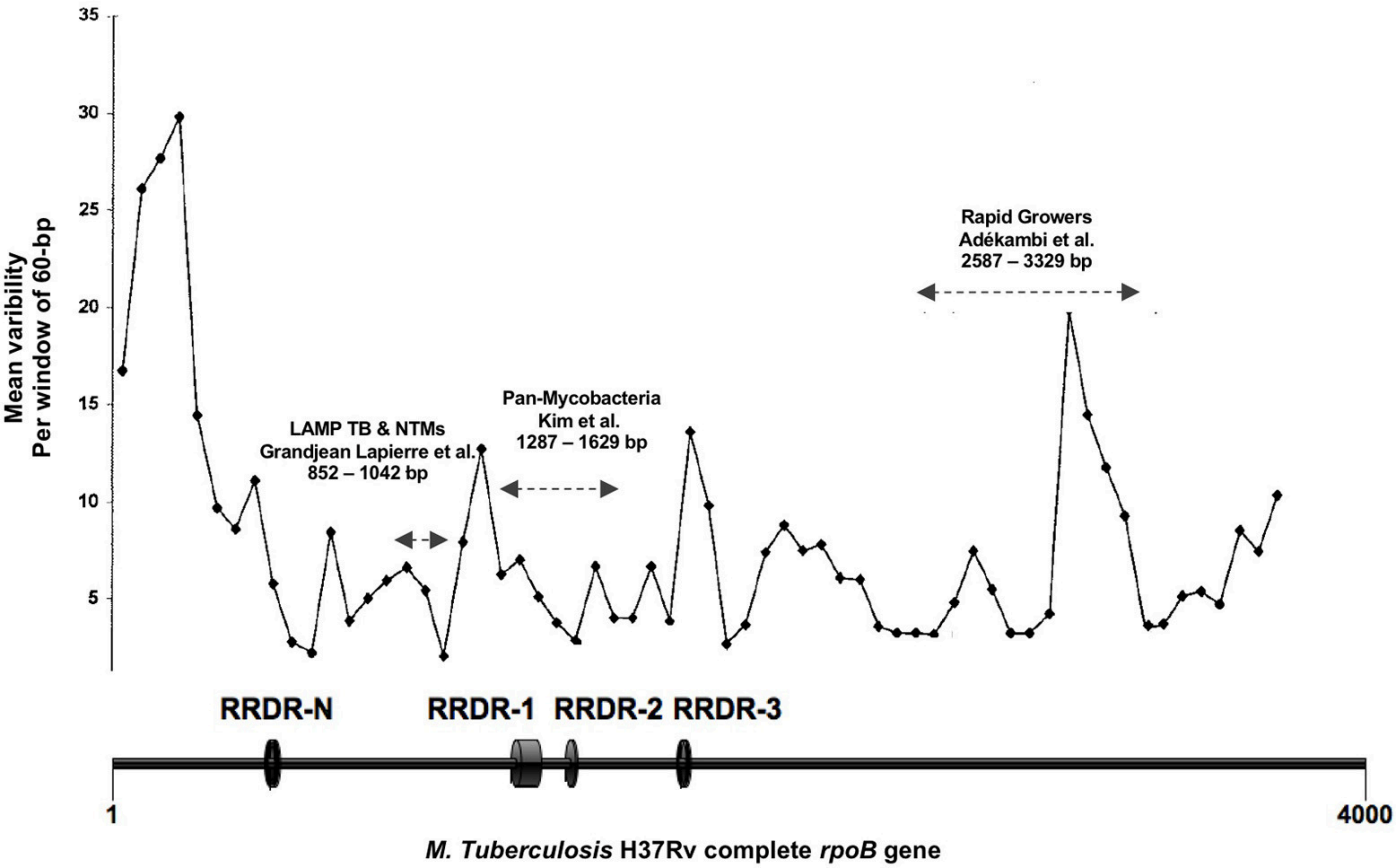
Figure adapted from Adékambi et al. (14). Representation of the mycobacterial *rpoB* gene regions inter-species variability expressed as number of single nucleotide polymorphism per 60 bp segments. RRDR, Rifampin resistance defining region; LAMP TB-NTMs, target sequence of the LAMP assay here presented; Pan-mycobacteria, target sequence for mycobacterial identification as described by Kim et al. (15); Rapid Growers, target sequence for rapidly growing mycobacteria identification as described by Adékambi et al. (14). Nucleotides positions with reference to *M. tuberculosis* H37Rv *rpoB* gene (GenBank accession no AL123456.3).

Whole genome reference sequences including those of the thirteen mycobacterial species and subspecies most frequently recovered from respiratory samples in the Institut Hospitalier Universitaire (IHU) Méditerranée Infection microbiology laboratory were obtained from GenBank database (Table 1). *rpoB* gene sequences were extracted and aligned using the *Mycobacterium tuberculosis* H37Rv genome as template (GenBank accession no AL123456.3). Hypervariable *rpoB* regions routinely sequenced for mycobacterial identification to the species level were not considered for the primer design analysis because of higher inter species genetic variability (14, 15). Rifampin resistance defining regions (RRDRs) known to contain single nucleotide polymorphism (SNP) responsible for drug resistance in *M. tuberculosis* were also excluded (Figure 1) (16). Inter-species *rpoB* gene highly conserved regions were analyzed using PrimerExplorer V5 software (Fujitsu, Tokyo Japan). Mutually exclusive sets of forward outer primer F3, backward outward primer B3c, forward inner primer FIP (F2-F1c), and backward inner primer BIP (B2c-B1) were identified. Inter-species nucleotide variability in F1c/B1 5′-terminal, F2/B2c 3′-terminal, and F3/B3c 3′-terminal sites was avoided to ensure optimal stringency in determinant primer annealing sites. When unavoidable such variability was tolerated in the F3/B3c 5′-terminal, F2/B2c 5′-terminal, F1c/B1 3′-terminal, and inner regions (8). Previously described loop primers and FIP/BIP primer spacers were not used (9). Individual primers specificity was assessed *in silico* using NCBI basic local alignment search tool (BLAST) against human and other bacterial, viral, or fungal DNA sequences. F1c/B1 and F2/B2c primer sequences were analyzed both independently and as a continuous fragment. Selected sets of LAMP primers were then tested on 10-fold serial dilutions of *M. bovis* BCG and *M. avium* subsp. *avium* DNA and the single primer set with highest analytic sensitivity was used for comprehensive assay performance analysis.

**Table 1:** *rpoB* pan-mycobacteria degenerate LAMP assay results.

		**Strain**	**Primer design source genomic sequence**	**Amplification result**			
				**Specific primers**	**Degenerate primers**		
**Mycobacteria**				**Pure culture**	**Pure culture**	**MGIT**	**Sputum**
MTB group	*M. tuberculosis*	H37Rv	AL123456.3	+/+/+	+/+/+	+/+/–	+/+/–
	*M. tuberculosis*	IHU clinical isolate	–	+/+/+	+/+/+	+/+/–	+/+/–
	*M. tuberculosis*	IHU clinical isolate	–	+/+/+	+/+/+	+/+/–	+/+/–
	*M. bovis* BCG	ATCC 35734	CP003494.1	+/+/+	+/+/+	+/+/–	+/+/–
	*M. bovis*	IHU clinical isolate	–	+/+/+	+/+/+	+/+/–	+/+/–
	*M. africanum*	IHU clinical isolate	FR878060.1	+/+/+	+/+/+	+/+/–	+/+/–
	*M. canettii*	IHU clinical isolate 1	FO203507.1	+/+/+	+/+/+	+/+/–	+/+/–
	*M. canettii*	IHU clinical isolate 2	–	+/+/+	+/+/+	+/+/–	+/+/–
*M.avium* complex	*M. avium* subsp *avium*	IHU clinical isolate	CP009614.1	+/+/–	+/+/+	+/+/–	+/+/–
	*M. avium* subsp *hominssuis*	IHU clinical isolate	AP012555.1	+/+/–	+/+/+	+/+/–	+/+/–
	*M. avium* subsp *paratuberculosis*	IHU clinical isolate	–	+/+/–	+/+/+	+/+/–	+/+/–
	*M. intracellulare*	ATCC 35847	CP003322.1	+/+/–	+/+/+	+/+/–	+/+/–
	*M. intracellulare*	IHU clinical isolate	–	+/+/–	+/+/+	+/+/–	+/+/–
	*M. chimaera*	IHU clinical isolate	CP012885.2	+/+/–	+/+/+	+/+/–	+/+/–
*M.abscessus* group	*M. abscessus* sensu stricto	ATCC 23006	CP009615.1	–/–/–	+/+/–	+/+/–	+/+/–
	*M. abscessus* sensu stricto	IHU clinical isolate	–	–/–/–	+/+/–	+/+/–	+/+/–
	*M. abscessus bolletii*	IHU clinical isolate	AP014547.1	–/–/–	–/–/–	–/–/–	–/–/–
	*M. massiliense*	IHU clinical isolate	CP003699.2	–/–/–	+/+/+	+/–/–	–/–/–
Other	*M. lentiflavum*	ATCC 51985	JN881350.1	+/+/+	+/+/+	+/+/–	+/+/–
	*M. lentiflavum*	IHU clinical isolate	–	+/+/+	+/+/+	+/+/–	+/+/–
	*M. simiae*	IHU clinical isolate	CP010996.1	+/+/+	+/+/+	+/+/–	+/+/–
	*M. kansasii*	ATCC 12478	CP006835.1	+/+/+	+/+/+	+/+/–	+/+/–
	*M. kansasii*	IHU clinical isolate 1	–	+/+/+	+/+/+	+/+/–	+/+/–
	*M. kansasii*	IHU clinical isolate 2	–	+/+/+	+/+/+	+/+/–	+/+/–
	*M. fortuitum*	IHU clinical isolate	CP011269.1	+/+/+	+/+/+	+/+/–	+/+/–
	*M. chelonae*	IHU clinical isolate	CP010946.1	–/–/–	+/+/+	+/+/–	+/+/–
	*M. malmoense*	IHU clinical isolate	MVHV01000002	+/+/+	+/+/+	+/+/–	+/+/–
	*M. xenopi*	IHU clinical isolate	LQQB01000145	+/+/+	+/+/+	N/A	N/A
	*M. gordonae*	IHU environmental isolate	LQOY01000155	–/–/–	+/+/+	+/+/–	+/+/–
**Actinomycetes**							
	*Tsukamurella* sp.	IHU clinical isolate	CP019066.1	–/–/–	+/+/–	+/–/–	+/–/–
	*Streptomyces* sp.	IHU clinical isolate	CP025407.1	–/–/–	+/+/–	+/–/–	+/–/–
	*Gordonia*	IHU clinical isolate	CP016594.1	–/–/–	–/–/–	–/–/–	–/–/–
	*Nocardia brasiliensis*	IHU clinical isolate	CP022088.1	–/–/–	–/–/–	–/–/–	–/–/–
	*Rhodococcus equi*	ATCC 33707	CM001149.1	–/–/–	–/–/–	–/–/–	–/–/–
	*Rhodococcus equi*	IHU clinical isolate	–	–/–/–	–/–/–	–/–/–	–/–/–
**Community and nosocomial acquired pneumonia pathogens**							
	*Staphylococcus aureus*	ATCC 25923	CP009361.1	–/–/–	–/–/–	–/–/–	–/–/–
	*Streptococcus pneumoniae*	ATCC 49619	CP025256.1	–/–/–	–/–/–	–/–/–	–/–/–
	*Haemophilus influenzae*	ATCC 10211	CP000672.1	–/–/–	–/–/–	–/–/–	–/–/–
	*Legionella penumophilla* subsp *pneumophilla*	ATCC 33152	CP015928.1	–/–/–	–/–/–	–/–/–	–/–/–
	*Legionella penumophilla* subsp *pneumophilla*	IHU clinical isolate	–	–/–/–	–/–/–	–/–/–	–/–/–
	*Escherichia coli*	ATCC 25922	AE014075.1	–/–/–	–/–/–	–/–/–	–/–/–
	*Pseudomonas aeruginosa*	ATCC 15442	CP007224.1	–/–/–	–/–/–	–/–/–	–/–/–
	*Pseudomonas aeruginosa* (mucoid variant)	IHU clinical isolate	–	–/–/–	–/–/–	–/–/–	–/–/–

*Semi-quantitative sensitivity analysis of rpoB Pan-Mycobacteria LAMP assay. Amplification results are presented for 10^4^, 10^3^, and 10^2^ cfu/mL standardized inoculum of every species for pure culture, sputum, and Mycobacteria Growth Indicator tube*.

### Study samples

Twenty recognized mycobacterial pulmonary pathogens comprising a combination of reference strains and clinical isolates from IHU reference mycobacteria laboratory, five aerobic actinomycetes rarely encountered in respiratory infections and six bacteria frequently responsible for community acquired and nosocomial pneumonia were used to evaluate LAMP assay performance (Table 1). Mycobacterial identification to the species level was confirmed using mass spectrometry and *rpoB* gene sequencing as per previously published protocols (14, 17). Bacterial pure cultures were obtained from Midlebrook 7H10, Colombia + 5% sheep blood or Buffered Charcoal Yeast Extract solid culture media per growth requirements. Standardized bacterial suspensions were prepared at 10^4^ CFU/mL, reported to be the detection limit of smear microscopy for *M. tuberculosis* (18). Ten-fold serial dilutions were also prepared to further assess LAMP assay analytic sensitivity. Sputum from healthy volunteer subjects and MGIT liquid culture media (Becton Dickinson, New Jersey, United States) were spiked with standardized bacterial concentrations to obtain the same bacterial inoculums.

### DNA extraction

Bacterial DNA was extracted from two mL of a pure culture suspension, two mL of supernatant from previously homogenized and centrifuged sputum and two mL of pellet from previously centrifuged MGIT culture medium bottle samples. After incubation in 200 uL G2^TM^ buffer (Quiagen, Hilden, Germany) and proteinase K for 10 min, study samples were lyzed using Fastprep^TM^ bead-beating benchtop homogenizer (MP Biomedical, California, United States) and heated to 70°C for 30 min. Extraction process was completed using EZ1^TM^ automated DNA kit (Quiagen, Hilden, Germany).

### LAMP reaction and amplification products detection

**Figure 2:**
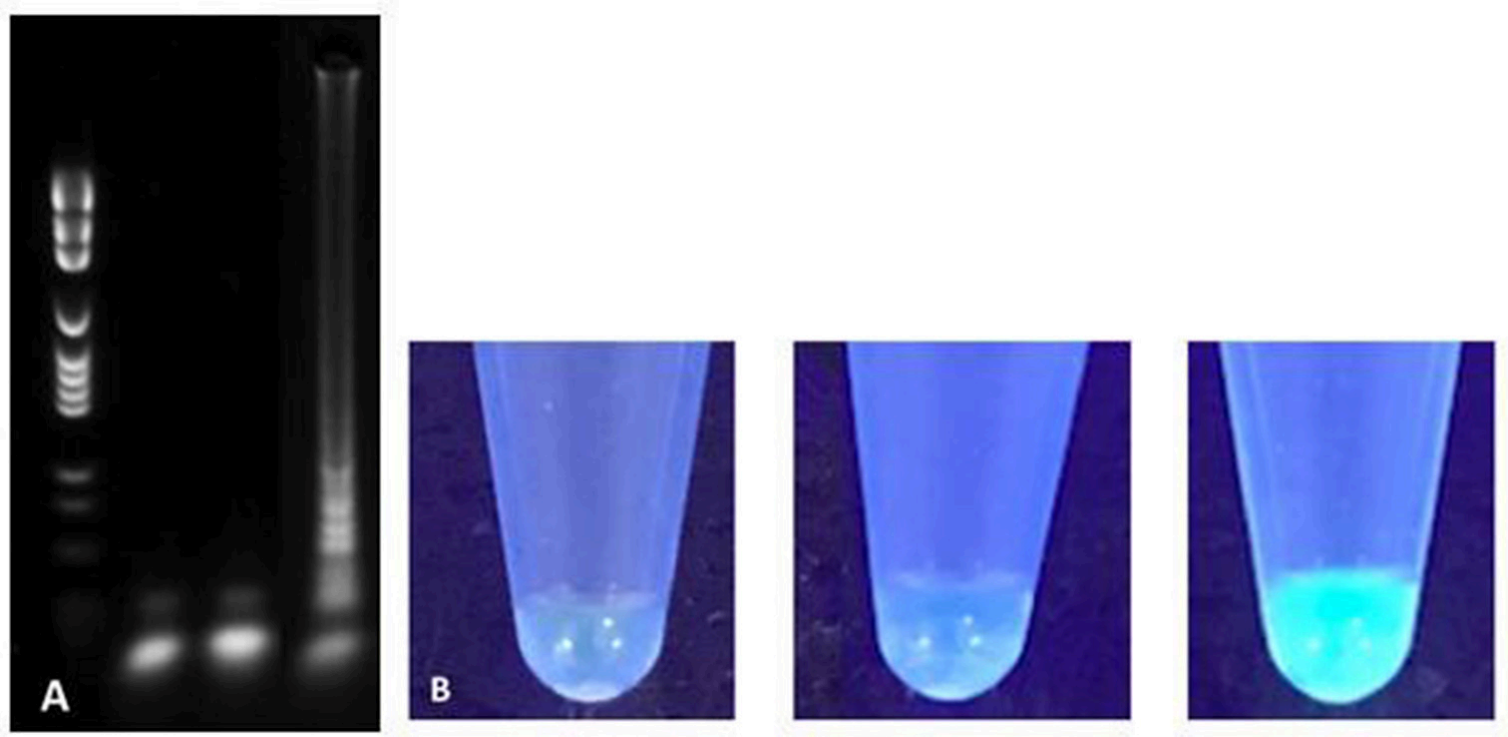
Agarose gel electrophoresis **(A)** and direct fluorescence **(B)** based detection of LAMP amplification products for H_2_O reagent control (left), *Escherichia coli* negative control (middle) and *M. tuberculosis* H37Rv (right).

LAMP reaction was performed using commercially available WarmStart^TM^ LAMP Kit (DNA and RNA) (New England Biolabs, MA, USA). Used reaction mix was as follows: 12.5 uL WarmStart^TM^ Lamp Master mix containing a *Bst* 2.0 polymerase (8,000 U/mL), 0.5 uL fluorescent dye, 1 uL target DNA, 1 uL pure DNA sample, 8.5 uL high quality PCR H_2_O, and 2.5 uL primer mix containing 1.6 uM FIP/BIP, 0.2 uM F3/B3, and 0.4 uM LoopF/B for a total reaction volume of 25 uL per reaction. Isothermal amplification was performed on a CFX96 Touch^TM^ cycler (Bio-Rad, CA, USA). Reaction temperature and time parameters were initially optimized using *M. bovis* BCG and *M. avium* subsp *avium* DNA template obtained from pure culture. A 35-min incubation period at 65°C followed by a 5-min polymerase inactivation phase at 85°C was found to be optimal. High-quality PCR H_2_O reagent control and *Escherichia coli* ATCC 25922^TM^ negative control were tested in parallel of every reaction. LAMP amplification products were detected by both agarose gel electrophoresis and direct fluorescence visual detection by two independent observers (Figure 2). Five microliter aliquots of LAMP products were electrophoresed for 35 min in 3% agarose gel and fluorescence detection was performed using SybeSafe^TM^ DNA staining. Visual detection was performed under UV lamp and relied on commercially available fluorescent dye included in LAMP master mix.

## Results

**Figure 3:**
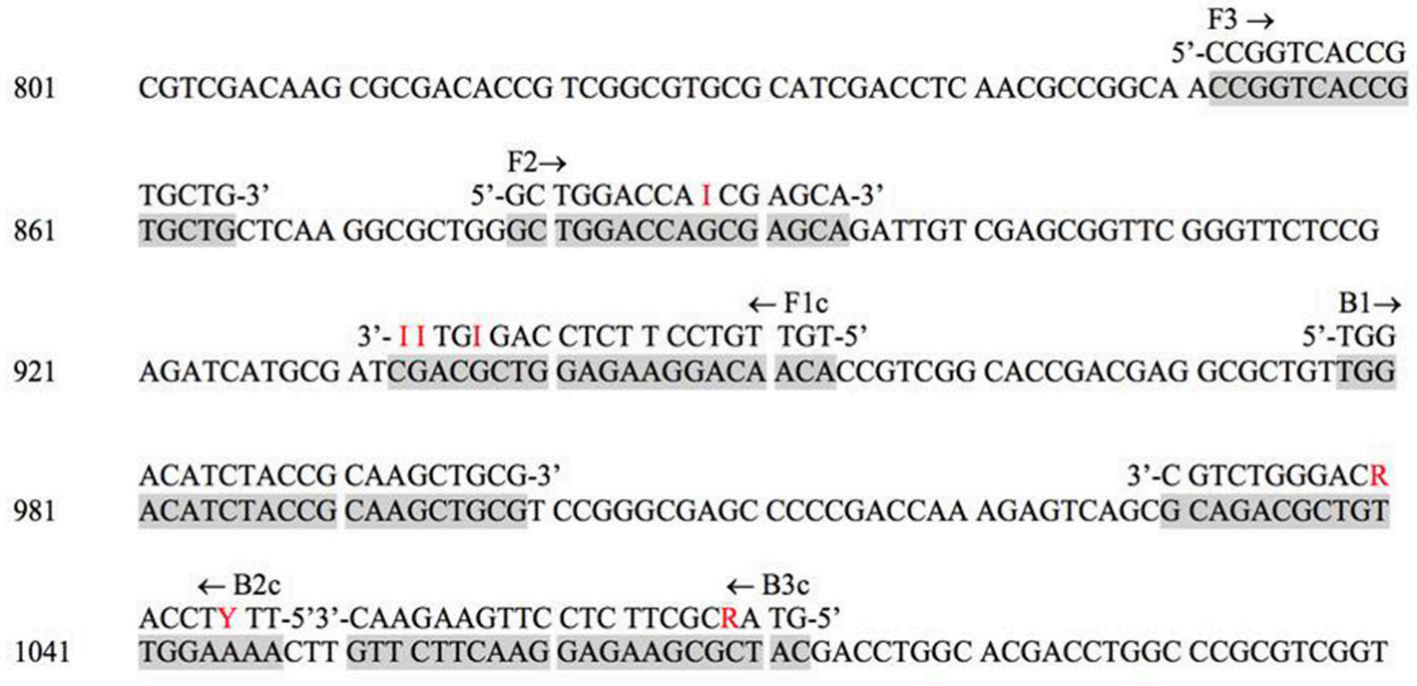
*M. tuberculosis* H37Rv reference strain *rpoB* target sequence with degenerate LAMP primer annealing sites. The primers are as follows: F3, forward; B3c, backward; FIP, forward inner (F1c and F2); BIP, backward inner (B2c and B1).

**Figure 4:**
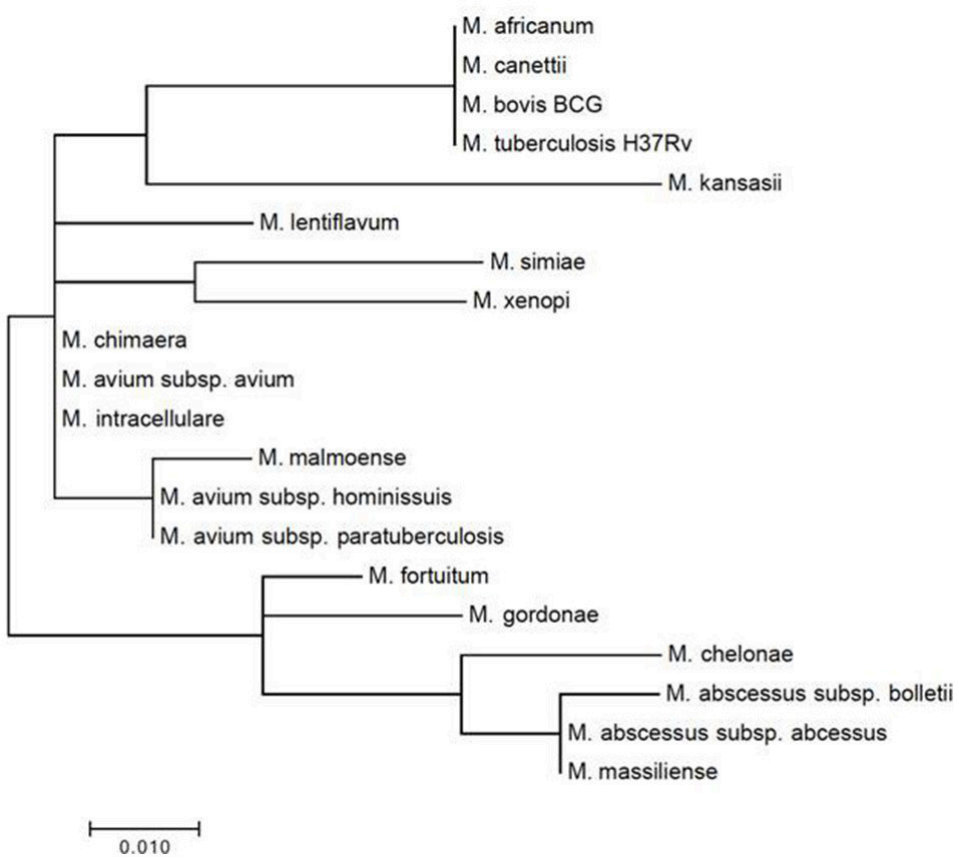
Mycobacterial inter-species phylogenetic relatedness analysis within the consensus degenerate *rpoB* 112 bps combined primer-targeted regions.

Three regions of the *M. tuberculosis* H37Rv *rpoB* gene (base pair positions 481–607, 852–1,072, and 1,970–2,065) were found to be highly conserved among species (Figure 1). Following *in silico* analysis, seven distinct sets of primers were selected for *in vitro* testing. Based on superior analytic sensitivity as demonstrated by consistent amplification of low DNA template inoculums of *M. bovis* BCG (10^1^ CFU/mL) and *M. avium* subsp*. avium* (10^2^ CFU/mL) in 2 mL a final set of primers was selected (Figure 3). F3; 5′-CCGGTCACCGTGCTG-3′, FIP 5′-TGTTGTCCTTCTCCAGIGTIIGCTGGACCAICGAGCA-3′, B3 5′- GTARCGCTTCTCCTTGAAGAAC-3′, BIP 5′-TGGACATCTACCGCAAGCTGCGTTYTCCARCAGGGTCTGC-3′. This set of LAMP primers includes a total of seven degenerate bases at selected unavoidable mutation sites in the B3c 5′-terminal, F2/B2c 5′-terminal, F1c 3′-terminal, and inner regions. Inter-species phylogenetic relatedness analysis within the *rpoB* 112 bps combined primer-targeted regions showed between 0 bp (*M. tuberculosis, M. bovis, M. bovis* BCG, *M. africanum*, and *M. canettii* sub-species of the *M. tuberculosis* complex) and 12 bps (*M. kansasii* and *M. bolletii*) variability between species (Figure 4). *In silico* analysis revealed aerobic actinomycetes to be genetically closely related to mycobacterial target species with *Tsukamurella* sp. (GeneBank accession No. CP019066.1) and *Streptomyces* sp. (GeneBank accession No. CP025407.1), respectively, sharing 7 and 13 of 112 bps with the *M. tuberculosis* H37Rv reference sequence within the LAMP primer annealing sites.

LAMP positive amplification reactions were successfully documented using gel electrophoresis and fluorescence direct visualization after a 35-min incubation. Concordance between both detection methods was absolute throughout the study. For all LAMP reactions presented here, reagent controls using water and negative controls using *E. coli* were consistently negative (Figure 2). To confirm the presence of mycobacterial DNA and rule out non-specific amplification due to primer auto-amplification which has been recognized as a challenge in LAMP assays optimization (manufacturer's communication, unpublished data), small fragments of amplification products of *M. bovis* BCG corresponding to bands with the highest molecular weight in the gel electrophoresis smear were extracted from agarose l and sequenced using MiSeq Next-Generation sequencer (Illumina, CA, USA). Short DNA fragments corresponding to LAMP targeted *rpoB* regions but excluding primer annealing sites were sequenced therefore confirming true positive results.

Native LAMP primers successfully amplified DNA extracted from pure cultures of various mycobacterial species including all of the *M. tuberculosis* complex, *M. avium* complex and *M. kansasii*. The inclusion of degenerate nucleotide bases in the LAMP primer set did not negatively impact detection threshold but further increased the number of mycobacterial species detected by the assay to all those included in this study except for *M. bolletii*. LAMP primers were shown to be highly specific for mycobacteria as no nosocomial or community-acquired pneumonia bacterial pathogens were amplified even after prolonging the reaction time to 60 min. Among selected aerobic actinomycetes with high similarity of the *rpoB* target sequence to that of the included mycobacterial species, only *Tsukamurella* sp. and *Streptomyces* sp. were amplified. When performed on DNA extracted from spiked sputum and liquid culture medium the LAMP assay yielded highly comparable species sensitivity and specificity. Only *M. bolletii* and *M. massiliense* could not be detected from spiked sputum samples. *M. xenopi* could not be tested in spiked sputum and MGIT samples because of insufficient growth. For most species, detection limit of the LAMP assay was 10^2^ CFU/mL when performed on pure culture and 10^3^ CFU/mL on sputum and MGIT broth (Table 1). Total duration of the LAMP assay was measured to be 90 min, including 45 min for extraction, 40 min for isothermal amplification, and 5 min for visual fluorescence detection. Required laboratory technician hands on time was 25 min.

## Discussion

We set up and evaluated the performance of a novel LAMP assay for consensus detection of mycobacteria from pure culture and spiked sputum and MGIT liquid medium samples. Previously published LAMP protocols for detection of mycobacterial species used conventional specific primer and targeted the 16S rRNA, *gyrB*, and insertion sequence genes (11–13). In this study, we designed the first LAMP assay to target the *rpoB* gene for mycobacterial detection. This is also the first description of a LAMP assay using primers containing degenerate nucleotide bases. Including degenerate bases in LAMP primers did not negatively impact analytical sensitivity but successfully increased the diversity of mycobacterial species detected by the assay. Mycobacterial inoculums as low as 10^3^ CFU/mL were detected in sputum and MGIT broth samples after 35 min. This detection threshold is higher than that of pure culture samples on which positive reactions were documented for inoculums as low as 10^2^ CFU/mL. As described in previous studies, extending LAMP reaction time might increase the sensitivity of the assay on sputum and MGIT broth samples but this was not a consistent finding in our study (11). Extending incubation time did not result in false positive reactions when testing a panel of non-mycobacterial respiratory pathogens. As reported in previous studies, false positive results were only observed with aerobic actinomycetes *Tsukamurella* sp. and *Streptomyces* sp., two species genetically closely related to mycobacteria (11). This specificity issue was considered of low clinical relevance as this these opportunistic microorganisms are rarely encountered in clinical lung disease. Whereas, previous studies evaluated the correlation between detection thresholds of LAMP and PCR for specific mycobacterial species, this was not performed in this study considering that no such assay exists for every single included species and that the LAMP assay described here was meant as pan-mycobacterial screening test.

Like other isothermal NAATS, our assay is rapid and technically simple to perform and only requires consumables, a heat block with constant temperature and a UV lamp for direct visual detection of results. It therefore has potential for rapid diagnosis of pulmonary mycobacteroses at the point-of-care. Despite our promising results and the many theoretical advantages of LAMP over PCR, this assay proved to have several limitations. Due to the structural complexity of the LAMP primer sets, accurate *in silico* tools to predict the incidence of primers secondary structures or sensitivity and specificity for target template are lacking. The resulting necessity of trial and error experimentation is time and resources consuming. Fluorescence-based visual detection of results can be equivocal especially in the presence of samples with lower inoculum. This ambiguity can easily be resolved by confirming results by gel electrophoresis although this detection method is not usually available outside the molecular biology laboratory. While concordance between gel electrophoresis and direct fluorescence as assessed by two independent observers was absolute in our study, un-experienced personnel in a point-of care setting might find the assessment of “low positive” samples challenging. Previously reported non-specific amplification was observed in multiple LAMP reactions. Indeed, positive reactions observed in reagent controls containing H_2_O and negative controls containing *E. coli* regularly occurred therefore invalidating the whole reaction. This was observed as frequently with specific and degenerate primers. DNA sequencing of LAMP amplification product confirmed true amplification of the mycobacterial target in reactions with adequate reagent (H2O) and specificity (DNA) controls.

These encountered challenges may explain why despite a multiplicity of published protocols and recognized medical, veterinary and environmental applications LAMP technology has failed to significantly scale-up at any level between point-of care settings and reference laboratories (19). Of major concern for routine laboratory services providers in conventional or point-of care settings are the visual result detection ambiguities and potential non-specific amplification in any given LAMP reaction. These may explain the absence of buy-in for LAMP technology in these contexts facing limited personnel training, high human resources turnover and the necessity for a streamlined sample workflow. Through increased reaction parameters standardization, automation could help overcoming some of these challenges as exemplified by the success of the WHO endorsed closed platform for LAMP-based detection of *M. tuberculosis* (20, 21).

In conclusion, we designed a novel LAMP assay targeting the mycobacterial *rpoB* gene and including degenerate primers for consensus detection of mycobacterial species from pure culture, sputum and MGIT broth samples. This assay showed good analytical sensitivity as well as mycobacterial species coverage and specificity. Despite these results and the advantages of LAMP over PCR, challenges such as result interpretation ambiguity and potential non-specific amplification precludes the routine use of this novel LAMP assay both in centralized and point-of care laboratory, as it was initially anticipated.

## Data availability statement

The datasets generated for this study are available from the corresponding author on request.

## Author contributions

SG study design, primer design and LAMP optimisation, and other laboratory experiments as well as manuscript writing. MD study design, prospective sample selection, and manuscript writing.

### Conflict of interest statement

The authors declare that the research was conducted in the absence of any commercial or financial relationships that could be construed as a potential conflict of interest.
